# Supplementary material for: Curated and harmonised transcriptomics datasets of interstitial lung diseases
Source: Data Brief. 2025 Oct 14;63:112139. doi: 10.1016/j.dib.2025.112139 (PMC12581653; doi:10.1016/j.dib.2025.112139)
Supplement: Supplementary file 1 [file mmc1.zip › Supplementary_material/RNA-seq/fastQC reports/GSE138283/SRR10212992_fastqc.html]

SRR10212992.fastq.gz FastQC Report 

FastQC Report

Fri 2 Feb 2024  
SRR10212992.fastq.gz

## Summary

- Basic Statistics
- Per base sequence quality
- Per sequence quality scores
- Per base sequence content
- Per sequence GC content
- Per base N content
- Sequence Length Distribution
- Sequence Duplication Levels
- Overrepresented sequences
- Adapter Content

## Basic Statistics

| Measure | Value |
| --- | --- |
| Filename | SRR10212992.fastq.gz |
| File type | Conventional base calls |
| Encoding | Sanger / Illumina 1.9 |
| Total Sequences | 70696718 |
| Sequences flagged as poor quality | 0 |
| Sequence length | 35-76 |
| %GC | 47 |

## Per base sequence quality

## Per sequence quality scores

## Per base sequence content

## Per sequence GC content

## Per base N content

## Sequence Length Distribution

## Sequence Duplication Levels

## Overrepresented sequences

| Sequence | Count | Percentage | Possible Source |
| --- | --- | --- | --- |
| CCACAAATTATGCAGTCGAGTTTCCCACATTTGGGGAAATCGCAGGGGTCAGCACATCCGGAGTGCAATGGATA | 480462 | 0.6796100492246331 | No Hit |
| CCCCACTACCACAAATTATGCAGTCGAGTTTCCCACATTTGGGGAAATCGCAGGGGTCAGCACATCCGGAGTGCA | 455718 | 0.6446098388895507 | No Hit |
| CCCACTACCACAAATTATGCAGTCGAGTTTCCCACATTTGGGGAAATCGC | 325216 | 0.4600156969097207 | No Hit |
| CACAAATTATGCAGTCGAGTTTCCCACATTTGGGGAAATCGCAGGGGTCA | 280863 | 0.397278696869634 | No Hit |
| CTCAGACCGCGTTCTCTCCCTCTCACTCCCCAATACGGAGAGAAGAACGATCATCAATGGCTGACGGCAGTTGC | 209727 | 0.296657335634732 | No Hit |
| CCTTAGGCAACCTGGTGGTCCCCCGCTCCCGGGAGGTCACCATATTGATG | 193561 | 0.2737906447085705 | No Hit |
| GTCGAGTTTCCCACATTTGGGGAAATCGCAGGGGTCAGCACATCCGGAGTGCAATGGATAAGCCTCGCCCTGGGA | 179537 | 0.2539537974025895 | No Hit |
| CTCCGTTTCCGACCTGGGCCGGTTCACCCCTCCTTAGGCAACCTGGTGGT | 165502 | 0.23410139067558977 | No Hit |
| CCCCTCCTTAGGCAACCTGGTGGTCCCCCGCTCCCGGGAGGTCACCATAT | 135355 | 0.191458675634702 | No Hit |
| CCAGGCTGGAGTGCAGTGGCTATTCACAGGCGCGATCCCACTACTGATCA | 119565 | 0.16912383400881495 | No Hit |
| GTCCCCCACTACCACAAATTATGCAGTCGAGTTTCCCACATTTGGGGAAA | 111634 | 0.15790549145435578 | No Hit |
| CCCTCCTTAGGCAACCTGGTGGTCCCCCGCTCCCGGGAGGTCACCATATT | 106836 | 0.15111875490457705 | No Hit |
| CACTACCACAAATTATGCAGTCGAGTTTCCCACATTTGGGGAAATCGCAG | 96605 | 0.1366470788643965 | No Hit |
| CTCAGGCTGGAGTGCAGTGGCTATTCACAGGCGCGATCCCACTACTGATC | 92522 | 0.13087170468083115 | No Hit |
| CCACTACCACAAATTATGCAGTCGAGTTTCCCACATTTGGGGAAATCGCA | 90310 | 0.12774284656325913 | No Hit |
| CTACCACAAATTATGCAGTCGAGTTTCCCACATTTGGGGAAATCGCAGGG | 84547 | 0.1195911244422973 | No Hit |
| CTGGAGTCTTGGAAGCTTGACTACCCTACGTTCTCCTACAAATGGACCTTGAGAGCTTGTTTGGAGGTTCTAGC | 83734 | 0.11844114177973579 | No Hit |
| GTGGCTATTCACAGGCGCGATCCCACTACTGATCAGCACGGGAGTTTTGA | 82163 | 0.11621897355970612 | No Hit |
| GCTCCGTTTCCGACCTGGGCCGGTTCACCCCTCCTTAGGCAACCTGGTGGTCCCCCGCTCCCGGGAGGTCACCAT | 75730 | 0.10711954124942548 | No Hit |
| CCTCCTTAGGCAACCTGGTGGTCCCCCGCTCCCGGGAGGTCACCATATTGATGCCGAACTTAGTGCGGACACCCG | 73012 | 0.10327494976499474 | No Hit |

## Adapter Content

Produced by FastQC (version 0.11.7)
